# The Binding Landscape of Serum Antibodies: How Physical and Mathematical Concepts Can Advance Systems Immunology

**DOI:** 10.3390/antib11030043

**Published:** 2022-06-23

**Authors:** József Prechl, Krisztián Papp, Ágnes Kovács, Tamás Pfeil

**Affiliations:** 1 R&D Laboratory, Diagnosticum Zrt, 1047 Budapest, Hungary; pkrisz5@gmail.com; 2 Department of Applied Analysis and Computational Mathematics, Eötvös Loránd University, 1117 Budapest, Hungary; agnes.kovacs@ttk.elte.hu; 3 ELKH-ELTE Numerical Analysis and Large Networks Research Group, 1117 Budapest, Hungary

**Keywords:** antibody, system, chemical potential, logistic function, differential equation

## Abstract

Antibodies constitute a major component of serum on protein mass basis. We also know that the structural diversity of these antibodies exceeds that of all other proteins in the body and they react with an immense number of molecular targets. What we still cannot quantitatively describe is how antibody abundance is related to affinity, specificity, and cross reactivity. This ignorance has important practical consequences: we also do not have proper biochemical units for characterizing polyclonal serum antibody binding. The solution requires both a theoretical foundation, a physical model of the system, and technology for the experimental confirmation of theory. Here we argue that the quantitative characterization of interactions between serum antibodies and their targets requires systems-level physical chemistry approach and generates results that should help create maps of antibody binding landscape.

## 1. Introduction

Our immune system is responsible for more than protecting us from pathogens. It also regulates the removal of our own molecules and cells once these are losing function due to ageing, attrition, or infection. It maintains a healthy balance with the myriad of microbes and viruses present in our bodies [1]. To carry out these functions, the system utilizes an intricate regulatory mechanism that tunes its potential for destruction over a very wide range [2,3]. The humoral adaptive immune system consists of cells (B cells) and soluble molecules (antibodies) and has the remarkable ability to generate an immensely diverse repertoire of its element by adjusting, amongst others, one critical factor of molecular interactions: affinity [4,5]. Together with albumin and other macromolecules antibodies, (Ab) creates a molecularly crowded environment in blood, where molecules are in constant interaction with each other. Because of the huge structural diversity of antibody binding sites, these interactions in the blood, and with all molecules and cells contacted by blood, the strength of binding interactions also spans a huge range. In this article we expand a conceptual framework, based on physics and B-cell differentiation, for the distribution and organization of antibody interactions, and argue that a recently developed quantitative serology technology is suitable for characterizing the proposed model.

## 2. Immunological and Physical Rules of the System: B Cells as Sensors and Effectors

Antibodies are present in three main forms in blood: as part of a receptor complex, the B-cell antigen receptor (BCR) or membrane immunoglobulin (mIg), with cellular signaling capacity [6,7,8]; in secreted, freely circulating form (this is what we usually refer to as serum antibodies) [9,10], and in receptor-bound form, attached to the immunoglobulin Fc receptors of cells [11,12,13]. The last form is responsible for effector functions and is not dealt with in this article but is also important in quantitative modeling of antibody homeostasis.

**Figure 1:**
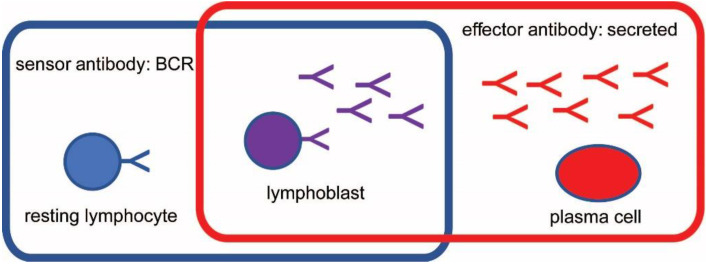
Categorization of antibodies based on sensor and effector function. The two functional types of antibodies, two corresponding cell types and the mixed type are shown. BCR, B-cell receptor.

Based on the form of antibody they express, there are three categories of B cells: (1) resting naïve B2 lymphocytes and memory B cells (MBC) display BCR but do not secrete Abs; (2) activated B1 cells, pre-plasmablasts, lymphoblasts express both surface and secreted Ab; (3) plasma cells, such as short-lived plasma cells (SLPC) and long-lived plasma cells (LLPC), only secrete antibodies. The second and third group together is also called antibody secreting cells (ASC). In accordance with these categories, these cell types function as antigen (Ag) sensors, as both sensors and effectors and as effectors only (Figure 1). A feedback mechanism based on antigen concentration and antibody engagement operates to generate sensors and effectors against all potential targets [8]. In short, the extent of antigen binding to BCR determines cell survival via signals delivered through the BCR [6]. Too much or too little BCR engagement leads to cell death, while the proper extent of BCR engagement initiates cell activation or cell survival. Activated B cells become lymphoblasts, with the ability to secrete antibody and yet depend on BCR signals for survival [14,15,16]. Terminally differentiated antigen secreting cells, plasma cells, do not depend on BCR signals [17,18,19] but produce secreted antibodies, which in turn reduce the concentration of target antigen [20]. As antigen is cleared the immune response retracts, short-lived effector cells (SLPC) die, and a new steady state equilibrium is established. Affinity maturation of antibodies changes the concentration of antigen required for a given extent of antibody engagement, therefore resulting in memory cells capable of more sensitive detection (sensor MBC) or more effective removal (LLPC) of antigen [20]. The new equilibrium allows MBC with increased sensitivity to survive, backing up the front line of secreted antibodies. Cycles of these events shape the lymphocyte repertoire and the theoretical space of all antibody interactions.

## 3. The Configuration Space of Serum Antibodies

From the medical and biological perspective, the humoral immune response takes place in various anatomical locations of the host: lymph nodes, spleen, bone marrow, blood, or periphery. The specialized structure of these tissues contributes to the development, differentiation and activation of B-cells and antibody secreting cells [4,21,22]. Nevertheless, all these tissues are physically connected, and while cell trafficking is regulated and cells are not allowed to go anywhere, secreted circulating antibodies do reach most tissues. Naïve and memory B cells are recruited into the pool of ASC by antigenic stimulation and co-stimulation by other cells [23,24]. There is a continuous supply of antibodies from ASC into the circulation, along with a continuous removal via immunoglobulin Fc receptors on immune effector cells [13,25,26]. This flow of antibodies maintains target antigen concentrations at levels defined by the immune system. Where antibodies are present, they continuously search for their highest affinity binding partner—in other words, for their lowest energy bound state. For a physical interpretation of the whole system of antibody interactions, it is reasonable to simplify the system, neglect anatomy, and introduce an abstract space instead: the antibody interaction space [27].

**Figure 2:**
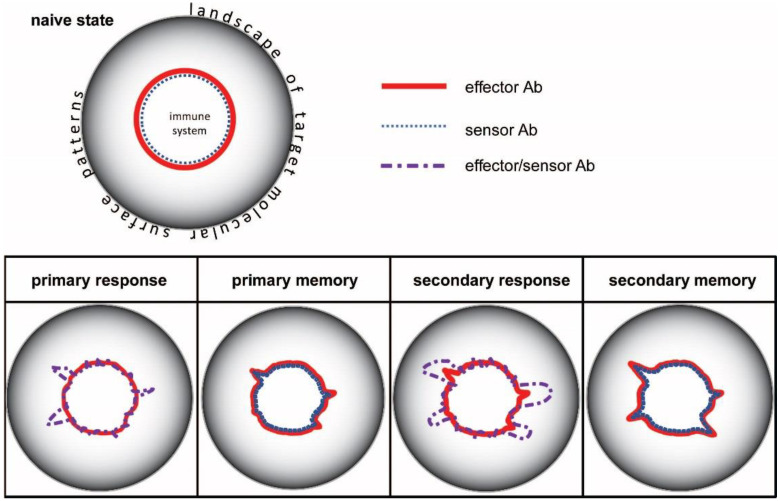
Immune responses displayed in configuration space of antibody interactions. Distance of the lines representing immune system boundary from the center corresponds to chemical potential.

This interaction space can be thought of as a coordinate system of chemical potentials. Chemical potential here refers to the ability of the system to contribute to the generation of Ab-Ag complexes. An Ab with given specificity can be identified by a vector pointing towards a given direction in the landscape of molecular targets (Figure 2). The chemical potential of the antibody is determined by the affinity (standard chemical potential), the concentration, and its thermodynamic activity coefficient (see later). In the center of the system is the generation of lymphocyte precursors, which develop into antibody secreting cells as they mature [27]. Within the boundaries of the system, cells generate a diverse repertoire of surface antibodies (B-cell receptors, BCR) that allows them to probe the complete antigen landscape or antigenome. In fact, BCRs are probing not whole molecules but rather patches of molecular surfaces called epitopes. We can think of the horizon of interaction space as the continuity of epitopes forming a canvas around the interaction space, as the landscape of target molecular surface patterns. Once a B-cell starts secreting an antibody, it will push the boundary of the system towards the recognized epitope, which is to an extent determined by its chemical potential (Figure 2).

**Figure 3:**
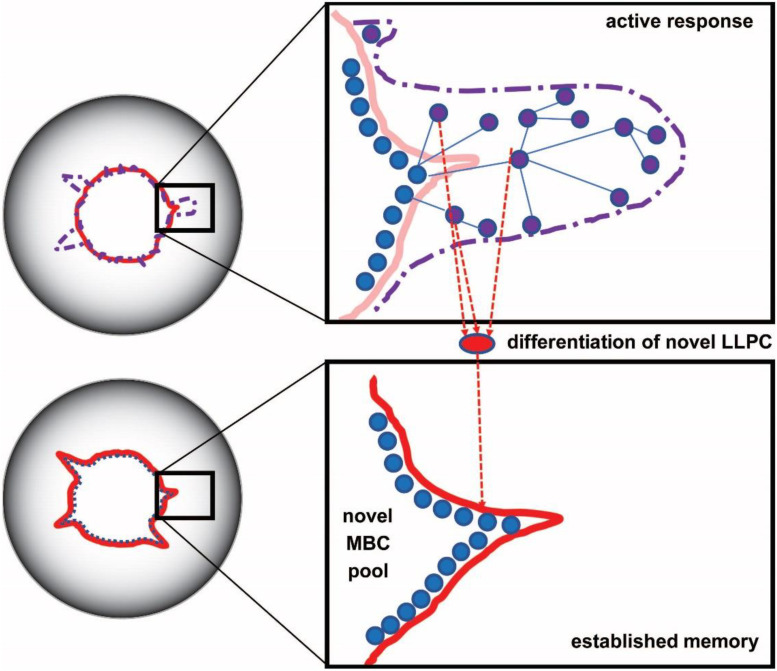
Expansion and affinity maturation of germinal center B cells displayed in configuration space. Naive and memory sensor B cells seed an active response, being activated via BCR. Somatic hypermutations generate random shifts in configuration space (blue lines between purple nodes), a selection of higher affinity mutants produce lymphoblasts. Secretion of antibodies with higher affinity appears as a protrusion of the interaction space towards the targeted antigen. Following an active immune response, the system retracts leading to a steady state with new borders, corresponding to LLPC and MBC with an affinity higher than original.

During an immune response, naïve and memory cells of the adaptive immune system are activated, expanded, and differentiated [28]. B cells in germinal centers undergo affinity maturation: somatic hypermutations introduce changes into antibody structure, followed by the selection of structural variants with higher affinity [4,5,29,30]. The process gives rise to genetically different new clones carrying antibodies with higher standard chemical potential. As long as the stimulus persists, germinal centers generate these new clones by cycles of random somatic hypermutation and selection. The result is the expansion of the system in the configuration space (Figure 2 and Figure 3): sensor-effector lymphoblasts start secreting antibodies and also increase their antibodies’ affinity by mutations [31].

As the stimulus is cleared by the immune response, most effector cells die and only memory cells remain. This corresponds to a retraction and reorganization in configuration space. It is important to note that the new boundary of the system is established by the negotiation between the host and the intruder: very harmful intruders will tend to leave a long-lasting and high affinity imprint, while softer attacks will have weaker effects [32,33]. Regulatory mechanisms in the host also cut back clones with potentially harmful autoimmune effects [34]. This negotiation results in a steady state, which entails the formation of networks that insert newly generated clones into a previously established architecture. The system of interactions optimizes itself: randomness is finally replaced by hierarchy and optimized antibody cross-reactivity networks. Activated cells will disappear, with resting lymphocytes and LLPC with adjusted affinity surviving (Figure 3).

The immune system is never totally at rest. It is the dynamism, the constant restructuring of this landscape by antigenic stimuli that maintains system architecture and adjusts the configuration space to the molecular environment. Therefore, whilst the overall hierarchy is expected to be governed by the laws of physics, shifting, and changing, locally active sites respond to the biological needs of the system.

## 4. Probing Serum Antibody Configuration Space: Quantitative Systems Serology

Understanding the underlying hierarchy and architecture of the network of antibodies has an immediate practical use: the design of serological assays with results that characterize this network. Current serological assays are standardized according to medical purposes, with the aim of establishing optimal cut-off values for diagnostic accuracy. The units obtained this way do not allow any kind of comparison of results, even for the identical antigen when different platforms are used, or different antibody isotypes are measured. The units are standardized but arbitrary with no biochemical meaning [35].

By using the configuration space model, we can identify the parameters that are required to describe such a system. Considering that serum antibodies are mixtures of molecules with a wide range of affinities against antigenic targets and a wide range of concentrations of each molecule, it is reasonable to assume that these parameters need to be estimated. We can probe this space by measuring the formation of antigen–antibody complexes in immunoassays and map the space by applying mathematical functions that model physical properties of the system.

The logistic function (also referred to as logistic equation, logistic growth curve, or Verhulst model) describes population growth with an exponential growth limited by maximum capacity of the system. The logistic function is the solution of the logistic differential equation, which describes rate of change against a given variable. While originally it was introduced for modeling growth in time, it is also used for modeling chemical reactions and antibody–antigen binding reactions [36]. In immunoassays we follow the increase of the equilibrium concentration of reaction products (Ab-Ag complexes) as a function of the logarithm of increasing concentrations of a reactant. Thus, the growth in this case is not in time but along an experimentally created concentration series. The time factor in these reactions can be omitted if the reaction is allowed to reach a point where concentrations of reaction components do not change any more, and equilibrium is reached.

For a reaction where we increase Ag concentration and follow the concentration of bound Ab (equivalent to measuring Ab-Ag complexes), we can rewrite the logistic differential equation

(1) $$\frac{dN}{dt}=r*N*\left(1-\frac{N}{K}\right)$$

where *N* is the number of entities and *K* is the capacity of the system for such entities, and r is the rate of exponential growth, as

(2) $$\frac{dAb_{b}}{dlog\left(Ag\right)}=r*Ab_{b}*\left(1-\frac{Ab_{b}}{Ab_{t}}\right)$$

where $Ab_{b}$ is bound antibody concentration, $Ab_{t}$ is total antibody, and Ag is total antigen concentration. We have previously described that $Ab_{b}$ is a logistic function of log(Ag) (see Supplementary Text S1 of [37]), which is the solution of this logistic differential equation. This means that the rate of change of generation of bound antibody while increasing Ag is determined by the actual bound antibody concentration, its relationship to the total antibody concentration, and the rate parameter.

We can simplify this expression by using the relative thermodynamic activity of antigen under equilibrium conditions. This activity is characterized by the thermodynamic activity coefficient of antigen $\gamma_{Ag}$, a coefficient that adjusts concentrations to relative thermodynamic activity a

(3) $$a_{Ag}=\gamma_{Ag}*Ag_{}$$

By normalizing, i.e., with $a_{Ag}=Ab_{b}/Ab_{t}$, Equation (2) becomes

(4) $$\frac{da_{Ag}}{dlog\left(Ag\right)}=r*a_{Ag}*\left(1-a_{Ag}\right)$$

The explicit solution of this differential equation is the function known to immunologists as the four-parameter logistic function or 4PL (see Appendix A) with a lower limit of zero. The 4PL can be used to estimate the affinity of a monoclonal antibody with known concentration [38]. However, the 4PL models an ideal binding curve, which may not reflect real binding that is modified by other interactions. To allow for an asymmetry in the binding curve and thereby take into account such intricate events, the five-parameter model, 5PL, was introduced (see Appendix A) [39].

**Figure 4:**
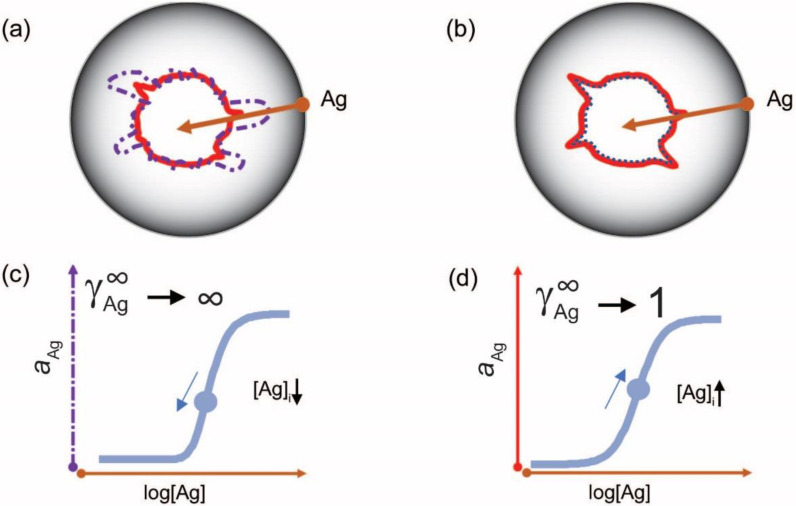
Probing the configuration space with antigen. Configuration space (**a**,**b**) can be probed (**brown arrow**) by measuring the changes of chemical potential of serum antibodies with the antigen of interest, using an immunoassay. Using antigen microspot titration key parameters of interaction, such as standard chemical potential and limiting activity coefficient, can be modeled by the Richards curve (**c**,**d**) parameters, including the determination of inflection point (**blue circle**) position. During an active immune response (**a**,**c**), the apparent affinity increases, as reflected by a decreased average standard chemical potential, and changes in clonal composition alter the limiting coefficient $\gamma_{Ag}^{\infty }$. A memory response (**b**,**d**) is characterized by optimized affinity and clonal heterogeneity. [Ag]_i_, antigen concentration at point of inflection.

**Figure 5:**
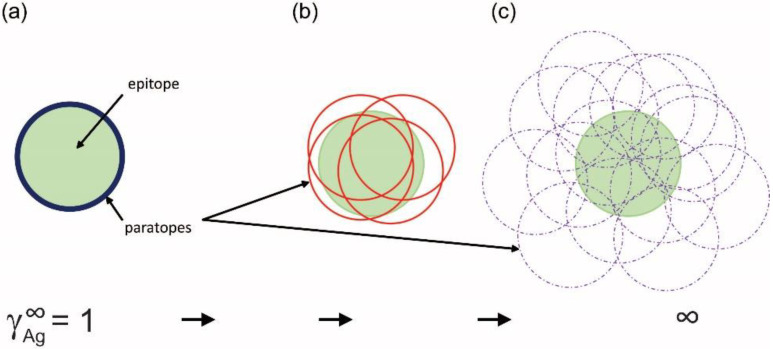
Interpretation of the limiting activity coefficient of antigen. The limiting thermodynamic activity coefficient reflects the contribution of epitope to binding by all antibody structures or the epitope-paratope fit in other words. Only the outlines of the paratope surfaces are shown (circles) to allow the visualization of overlaps. (**a**) A monoclonal antibody paratope-epitope fit is shown for comparison. Memory formation (**b**) reduces surviving clones to minimal optimal binders, while during an active immune response (**c**) several structurally distinct antibodies co-exist and compete for binding.

However, while the 5PL is the solution of a modified differential equation, it is less suitable for the description of a system’s behavior because of its parametrization. First order differential equations define relationships between functions representing physical quantities and their derivatives, the latter representing rates of change of the physical quantity. In an immunoassay, the physical quantity is the amount of antigen–antibody complex (or bound antibody) formed during the assay once equilibrium is reached. The rate of change in this quantity, while changing reaction conditions by titrating antibody or antigen, is the derivative of the function that relates the amount of complex formed to the logarithm of the titrated component. We proposed the use of the generalized logistic model (GL) or Richards growth model [40,41] instead of 5PL [37], because the Richards growth function, like the 5PL, is the solution of the differential equation

(5) $$\frac{da_{Ag}}{dlog\left(Ag\right)}=\frac{r}{\nu }*a_{Ag}*\left(1-{\left\{a_{Ag}\right\}}^{\nu }\right)$$

but is parameterized in a way that the logarithm of the inflexion point [*Ag*]*_i_* is one of the parameters. This differential equation implies that besides the activity coefficient of antibody and the rate parameter, the rate of generation of bound antibody while increasing *log*(*Ag*), is determined by a power of the activity coefficient. The exponent in the power expression ν is a parameter that modifies the influence of the ratio $\frac{Ab_{b}}{Ab_{t}}$ on the rate of growth of bound antibody. The reason for this modification is the changing behavior of antibodies at different antigen concentrations. This parameter introduces asymmetry into the sigmoid binding curve in a way that is more suitable for the description of the system. We propose that ν is related to a special activity coefficient, $\gamma_{Ag}^{\infty }=1/\nu ,$ which defines Ag thermodynamic activity at infinite Ab dilution and is determined by the composition of antibodies in the total pool (Figure 4 and Figure 5).

By fitting the Richards curve to experimental binding data from Ag and Ab titration experiments we can obtain the parameters that are suitable for the quantitative characterization of serum Ab. One of these is the antigen concentration at the point of inflection [Ag]_i_ and the other is the limiting activity coefficient (Figure 4) [37]. The first is an estimate of the apparent equilibrium dissociation constant (determined by average standard chemical potential) of the antibodies bound to the Ag. The second characterizes the hierarchy of antibodies bound to the Ag. A proof-of-concept study by our group, utilizing antigen microspot titration on protein arrays, suggests that this approach provides quantitative results suitable for comparing distinct isotypes or different antigens [37]. The mapping of these values to collections of structurally related epitopes could serve as a starting point for describing the serum antibody binding landscape.

## 5. Consolidation and Steps towards Systems Serological Mapping of Immunity

Our model of configuration space and mathematical approach to specific serum antibody measurement builds on idea that B cells can only remain in the resting state if they receive the appropriate signals via the BCR [8]. This is called tickling: a certain degree of BCR engagement between under- and overstimulation [42]. In order to remain in this state, B cells can modulate antibody variable region (affinity) and constant region (isotype and glycosylation). If these changes result in the adjustment of antigen binding in a way that the B cell collects survival signals then, following differentiation, it becomes a resting B cell again. If these effector properties are successfully selected by class switching, then the antigen concentration is properly set for the purposes of the particular B cell clone.

The equation we propose for use, the GL instead of 4PL and 5PL, estimates the average and the distribution of affinities of antibodies against a given antigen. Antibody-mediated clearance should depend on the generation of immune complexes, which itself is determined by the affinity of antibodies. The binding of antibodies to antigens and the clearance of the formed complexes should be harmonized, otherwise either immune complexes would accumulate (high affinity, low clearance, as it happens under some pathological conditions) or would unnecessarily remove antibodies and antigen (low affinity, fast clearance). The overall balance can be kept if the immune system tunes clearance to match affinity by switching isotypes to enhance FcR binding and complement activation when affinity increases. This harmonization shapes the binding landscape, which is modeled by the proposed configuration space.

The equation we propose addresses serum antibody measurements and thereby systemic immune responses. Because lymphatic circulation drains into the blood via sites of lymphocyte differentiation and activation, the lymph nodes and systemic immune responses have access to practically all antigens in the host. The other way round, antibodies in the bloodstream also reach lymphoid organs. It is in the lymphoid organs that affinity maturation takes place and adjusts distribution of affinities. Otherwise, at sites where the flow of immune complexes is unidirectional (out of the host), like mucosal surfaces, we do not expect to have direct effects on the equation. Consequently, we also do not expect that the systemic configuration space model is applicable to mucosal surfaces.

The key message of our article is that the binding landscape of serum antibodies cannot be approached as the simple sum of individual, independent interactions. The word binding is rather meaningless unless we identify conditions and quantify interaction energy. Blood plasma is crowded with macromolecules, with a significant contribution from circulating antibodies. The conditions are therefore defined by the composition of antibodies by the intricate cross-reactivity network of antibodies, their structures, and concentrations. Most of the immunological studies have been directed towards defining how an active immune response happens. Molecular biology helped us clone, sequence, and recombinantly express monoclonal antibodies. Structural biology allowed the characterization of antibody structures in detail. Now it is time to organize this information into a complex biological system. We propose that instead of examining the active phase of an immune response, the characterization of landscape of serum antibody binding in steady state is a better goal from the point of view of physics.

The ability to quantitatively characterize and map serum antibody binding to vast collections of antigens can open several possibilities. Via the standardization of simplex measurements, we could generate comparable binding data from quantitative immunoassays and integrate that into epitope databases. Epitope databases would develop into quantitative databases in terms of incorporating binding strength data. By generating antigen arrays with whole molecule antigens, peptides, and modified random peptides suitable for binding strength quantitation, we can attempt to create complete maps of serum antibody binding landscape. By the selective detection of isoforms IgG, IgA, IgM, and IgE, a further dimension, related to biological effects, can be introduced into the database. In the long term, such quantitative maps of individual’s Ab interaction spaces should become the foundations for immunodiagnostics and therapeutics as well. Our model provides a theoretical framework of systems-level physical approach to the functioning of adaptive immunity. Experimental testing will be necessary to see how and when and with what accuracy the model can be applied to map the antibody landscape of a living organism, especially with regard to the relationship of MBC and secreted antibodies, the effects of antigen and antibody multivalency, and the possibilities of merging quantitative binding data with BCR repertoire sequencing.

## Data Availability

Not applicable.

## Appendix A

Comparison of logistic functions

Basic function:

$$y=\frac{1}{1+{\left(\frac{x}{x_{i}}\right)}^{-r}}$$

**Table A1:** Comparison of parametrization, equations and graphical appearance of logistic functions. Representative curves in the diagrams use identical [Ag]_i_ and r, and the indicated ν values. diff. eq., differential equation. To help the comparison of the three logistic functions, the minimum and maximum values are set to 0 and 1, respectively. This simplifies the functions, though the number of parameters is less than suggested by the name. This normalization also allows to treat y as the relative thermodynamic activity of Ag, instead of the concentration of bound antibodies. The basic function is modified as implicated in the table to obtain the respective functions: four-parameter logistic (4PL), five-parameter logistic (5PL), and generalized logistic (GL) or Richards function. As the functions show, compared to the 4PL, the 5PL function introduces parameter ν as the exponent 1/ν of the denominator. The generalized form, in addition to this change, also introduces ν as a multiplying factor of the ratio of antigen concentration [Ag] and antigen concentration at inflection point [Ag]i with power −r.

	4PL	5PL	GL
x = [Ag] x_i_ = [Ag]_i_	$a_{Ag}=\frac{1}{1+{\left(\frac{x}{x_{i}}\right)}^{-r}}$	$a_{Ag}=\frac{1}{{\left\{1+{\left(\frac{x}{x_{i}}\right)}^{-r}\right\}}^{\frac{1}{\nu }}}$	$a_{Ag}=\frac{1}{{\left\{1+\nu {\left(\frac{x}{x_{i}}\right)}^{-r}\right\}}^{\frac{1}{\nu }}}$
x = log[Ag] x_i_ =log[Ag]_i_	$a_{Ag}=\frac{1}{1+e^{-r\left(x-x_{i}\right)}}$	$a_{Ag}=\frac{1}{{\left\{1+e^{-r\left(x-x_{i}\right)}\right\}}^{\frac{1}{\nu }}}$	$a_{Ag}=\frac{1}{{\left\{1+\nu e^{-r\left(x-x_{i}\right)}\right\}}^{\frac{1}{\nu }}}$
diff. eq.	$\frac{da_{Ag}}{dlog\left[Ag\right]}=ra_{Ag}\left(1-a_{Ag}\right)$	$\frac{da_{Ag}}{dlog\left[Ag\right]}=\frac{r}{\nu }a_{Ag}\left(1-{\left\{a_{Ag}\right\}}^{\nu }\right)$	
f(x)			
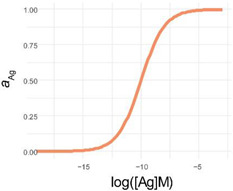
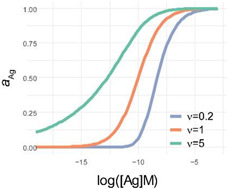
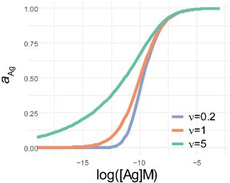
$y=loga_{Ag}$	$loga_{Ag}=-log\left(1+e^{-r\left(x-x_{i}\right)}\right)$	$loga_{Ag}=-\frac{1}{\nu }\log\left(1+e^{-r\left(x-x_{i}\right)}\right)$	$loga_{Ag}=-\frac{1}{\nu }\log\left(1+\nu e^{-r\left(x-x_{i}\right)}\right)$
log f(x)			
